# Network-based risk assessment of ship-mediated dispersal of non-native species across Chilean and international ports

**DOI:** 10.1038/s41598-025-15482-y

**Published:** 2025-08-20

**Authors:** Javier Pinochet, Reinaldo Rivera, Antonio Brante

**Affiliations:** 1 Departamento de Ecología, Facultad de Ciencias, Universidad Católica de la Santísima Concepción, Concepción, Chile; 2 Centro de Investigación en Biodiversidad y Ambientes Sustentables (CIBAS), Universidad Católica de la Santísima Concepción, Concepción, Chile; 3 Millennium Institute of Oceanography (IMO), Universidad de Concepción, Casilla 160-C, Concepción, Chile

**Keywords:** Maritime transport, Marine invasion, Network, Environmental similarity, Conservation biology, Invasive species

## Abstract

Marine biological invasions threaten global biodiversity, making it essential to identify high-risk areas for effective management and prevention. This study assesses the risk of non-native species (NNS) dispersal through maritime transport using network analysis to examine connectivity patterns among Chilean and international ports, complemented by an environmental similarity approach for estimation. The results highlight San Antonio, San Vicente, and Concepción Bay as key nodes within the national network, facilitating maritime traffic redistribution and linking the Central Chile and Araucanian ecoregions, identified as critical corridors for NNS introduction and spread. In contrast, secondary ports such as Corral and Mejillones have limited connectivity and play minor roles in the network. Internationally, Chilean ports maintain frequent connections with global hubs such as Panama and Shanghai, emphasizing their role in global maritime traffic. Procrustes analysis reveals strong environmental similarity between connected regions, suggesting that shared conditions enhance NNS survival and establishment. Alluvial diagrams and network illustrate high-risk routes and port, aiding in the identification of critical areas for monitoring and management. This study underscores the importance of integrating network analysis with environmental data as a key tool for assessing invasion risk, prioritizing strategic areas, and strengthening preventive strategies.

**Supplementary Information:**

The online version contains supplementary material available at 10.1038/s41598-025-15482-y.

## Introduction

The introduction of non-native species (NNS) is the active or passive displacement of organisms beyond their natural geographic range by human-mediated vectors, with potential ecological, economic and health impacts^1^. In marine systems, two main vectors are recognized: ballast water carried by ships and the biofouling that develops on hulls and niche areas^2–6^.

Ballast water has been subject to binding international control since the entry into force of the Ballast Water Management Convention in 2004^1^, whereas biofouling remains governed only by non-binding guidelines and broader invasive species recommendations that are still under development^2,3^. Functionally, ballast water discharge releases organisms in a single, event-based pulse when tanks are emptied at the destination port^4,5^; in contrast, hull biofouling transports live communities that can detach or reproduce continuously throughout an entire voyage, creating a persistent propagule supply at every stopover^6,7^. Case studies from the North Sea^8^, and the Australian coast^9^ confirm that areas with high environmental similarity and maritime connectivity are particularly vulnerable to biological invasions. These examples underscore the global relevance of the problem and the need to consider both international and national dynamics when evaluating invasion risk. The risk posed by ballast water was first acknowledged in 1973 through MARPOL 73/78. However, the International Maritime Organization (IMO) issued preliminary guidelines in 1991, revised them in 1997, and in 2004, adopted the BWM Convention, which set international standards to prevent the spread of harmful aquatic organisms^1,10^.

Chile has not yet ratified the BWM Convention; however, since 2012 the Maritime Authority (DIRECTEMAR) has required every international vessel to submit a Ballast Water Reporting Form and certify open-ocean exchange under Regulation D-1 (at least 200 nautical miles from the coast and 200 m depth) via Circular A-51/002^11,12^. Regarding biofouling, Circular A-52/007 allows underwater cleaning of hulls on domestic or international routes only when navigation safety is at risk, without setting limits on biofouling coverage leaving a potential pathway for NNS introduction^13^.

Scientific evidence confirms the arrival and circulation of high-risk taxa through both vectors. A ballast-water survey of twelve vessels recorded high concentrations of toxic phytoplankton, including *Pseudo-nitzschia delicatissima* and *Dinophysis acuminata*, together with viable zooplankton dominated by copepods (*Paracalanus cf. indicus* and *Oithona* spp.) that showed survival rates above ninety per cent in tanks at 12–22 °C and pH 6.3–8.6^14^. Biofouling samples from international ships berthed in Talcahuano port revealed the ascidian *Asterocarpa humilis* (native to New Zealand), the bryozoan *Bugulina flabellata* and the ascidian *Ciona robusta*, the latter with high haplotype diversity, indicating strong propagule pressure^15^. *Asterocarpa humilis* has extended its range by more than 2,000 km, from Antofagasta to the Araucanian ecoregion, through hull transport and subsequent establishment on mussel-farming ropes; this pattern confirms the combined role of port connectivity and environmental similarity in its spread^16^.

Despite existing measures, international shipping and local fishing-and-tourism fleets continue to facilitate NNS dispersal at local and regional scales, linking mainland Chile with island territories and remote areas such as Antarctica^6^. International traffic has been widely studied^17^, whereas domestic traffic, including commercial and tourist vessels, has received less attention. Interaction between these dynamics may intensify invasion processes by connecting global and local spatial scales and bridging ecoregions and broader biogeographic realms. Along the Chilean coast, 51 non-indigenous, cryptogenic or range-expanding species have been recorded^18,19^ although this scenario is probably underestimated because large-scale surveys are lacking and sampling effort remains limited in some regions^18^. Local surveys in Biobío ports have documented NNS on the hulls of vessels operating between national terminals^15,16,20–22^.

In this context, similar environmental conditions between source and destination ports, continuous vessel circulation and the absence of natural barriers combine to increase invasion likelihood. Vulnerability is heightened because maritime transport handles about ninety per cent of Chile’s foreign trade^23,24^ in 2023, thirty-nine ports received 6,557 international vessels that collectively discharged a total of ten million tons of ballast water^25^. Because maritime traffic underpins both ballastwater discharge and hullfouling transport, our analysis focuses on traffic patterns as a first step to identifying potential highrisk routes. Network analysis, widely used in research, conceptualizes ports as nodes and shipping routes as edges with specific attributes, thereby facilitating the assessment of biological invasion risks^26,27^. In contrast, species-distribution models (SDM) primarily focus on environmental suitability^28^, while network analysis explicitly incorporates connectivity patterns, allowing the identification of high-risk areas for species introductions This study is the first in Chile to evaluate potential routes for the introduction and spread of species and propagules between ports and ecoregions. We do not quantify ballastwater or biofouling loads; instead, we infer relative risk from network centrality and environmental similarity. Our objective is to identify the Chilean ecoregions, routes and ports most susceptible to biological invasions along the coast using a combined approach based on network analysis and environmental similarity.

## Results

### Connectivity and dispersion in national and international maritime traffic network

**Fig. 1:**
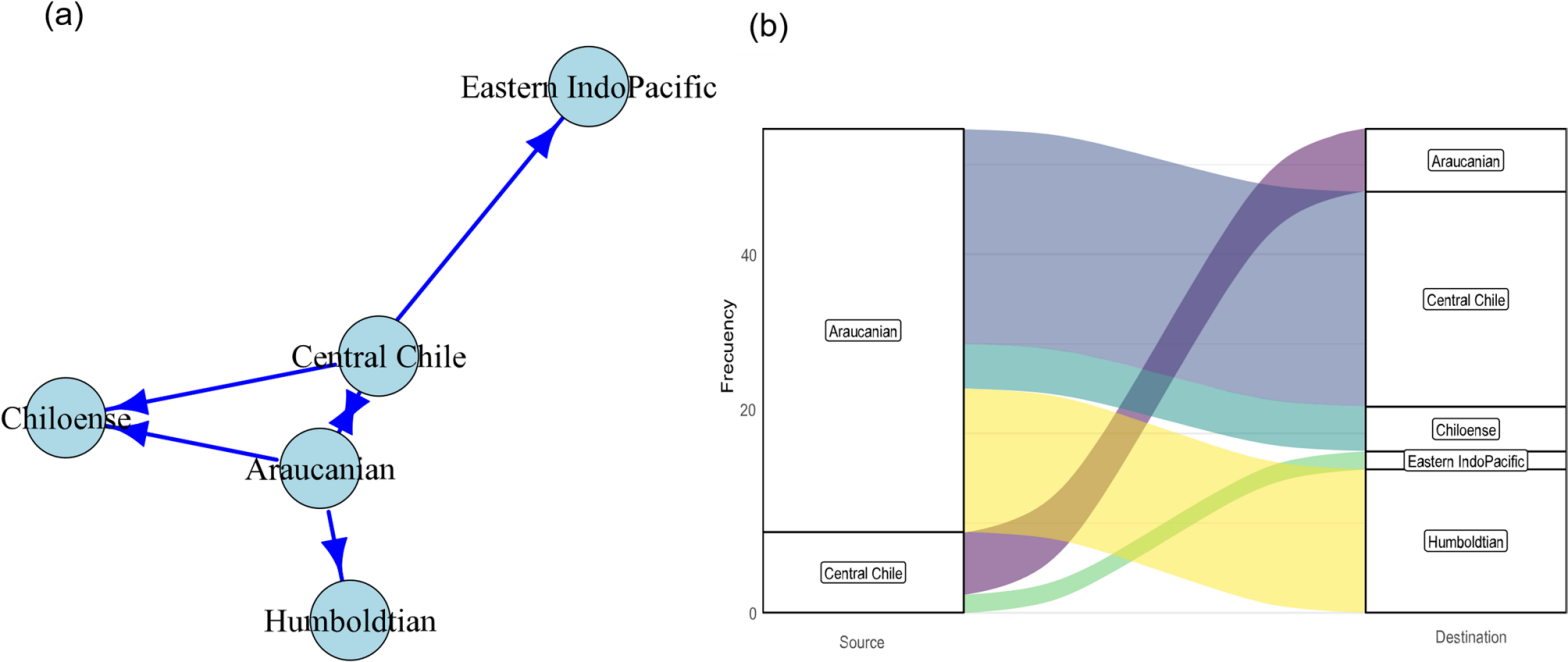
(**a**) Network of vessels between marine ecoregions of Chile. The size of the nodes represents the strength of connectedness. (**b**) Alluvial diagram relating the source ecoregions with the destination ecoregions outside Chile. The connection width is proportional to the number of events. Figures done using R (https://www.r-project.org).

Network analyses identified key maritime connections between Chilean ecoregions. At the national level, Central Chile and Araucanian ecoregions stand out as the most interconnected, with vessel flows directly linking them (Fig. 1a). These ecoregions concentrate high traffic, acting as major corridors within the national maritime network. All centrality indices confirm the relevance of these ecoregions in the network, showing high connection strength (Table S1), as well as a high level of similarity in environmental conditions, indicating that the habitats share comparable environmental characteristics (Table S2). In contrast, the Humboldtian and Chiloense ecoregions exhibit lower connectivity, with reduced flows suggesting a secondary role within the national network. By contrast, maritime event flows within Chile identify the Araucanian and Central Chile ecoregions as the primary sources and destinations. This reflects a high level of connectivity primarily between these ecoregions, as well as between the Araucanian and Humboldtian regions (Fig. 1b).

**Fig. 2:**
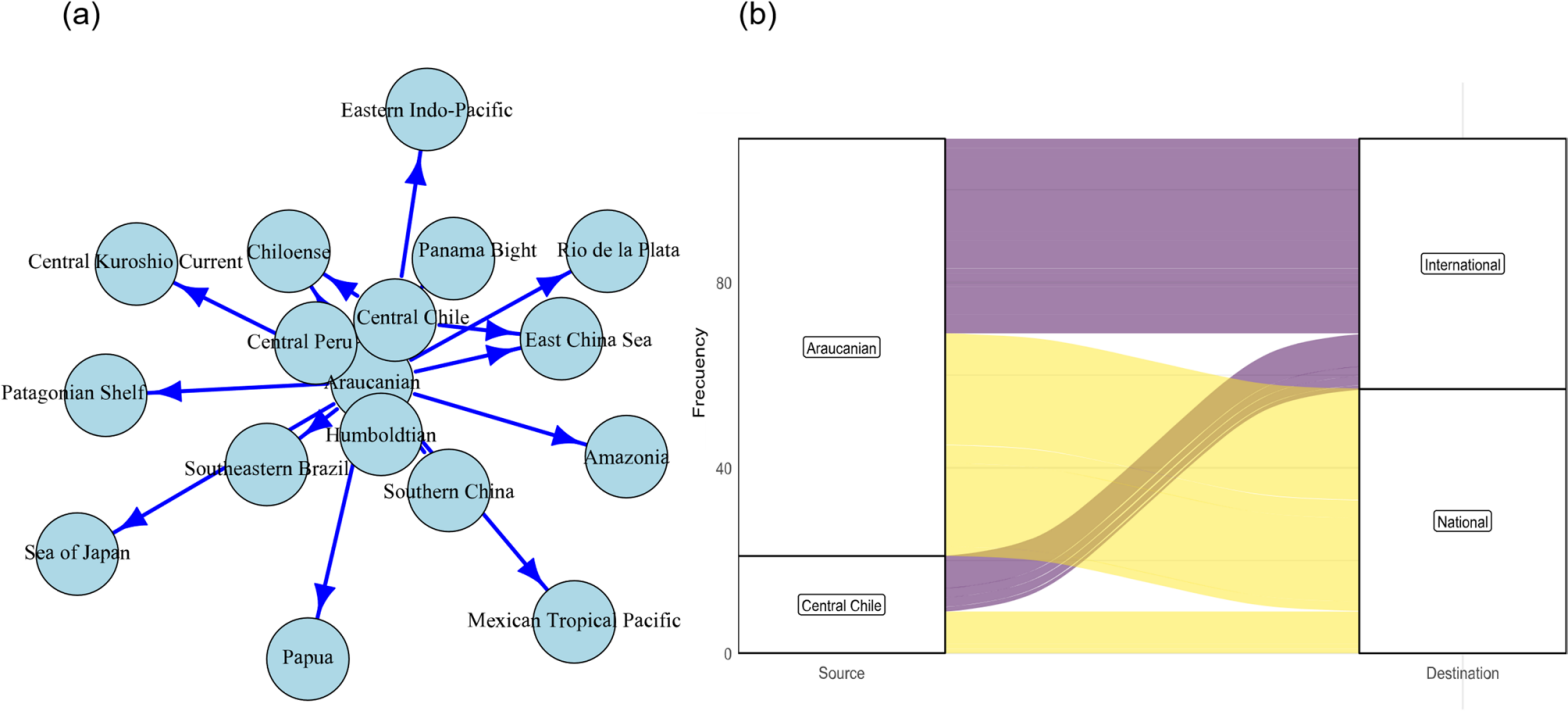
(**a**) Network of vessels between marine ecoregions of Chile and internationally. The size of the nodes represents the strength of connectedness. (**b**) Alluvial diagram relating departure ecoregions to destination ecoregions within Chile. Connection width is proportional to the number of events. Figures done using R (https://www.r-project.org).

At the international level, the Central Chile and Araucanian ecoregions dominate these interactions, maintaining frequent flows to regions like the Eastern Pacific and the Amazonia (Fig. 2a). These ecoregions stand out for their high connectivity, functioning as critical nodes in the global maritime traffic exchange. At the international level, centrality indices (Table S3) reinforce the importance of these ecoregions, showing high values in metrics such as degree and strength. In contrast, ecoregions like the Humboldtian play a limited role in international connections, with significantly smaller flows. At the environmental level, Central Chile and the Araucanian region exhibit a high degree of environmental similarity with ecoregions located in Asia, the North Pacific, and the South Atlantic (Table S4). This environmental convergence suggests the presence of analogous bioclimatic envelopes across these geographically distant areas, potentially promoting biotic exchange.

The connection flows between Chilean ecoregions and their international destinations reveals that the Central Chile and Araucanian ecoregions emerge as the main origin nodes, with frequent connections with international ports (Fig. 2b). The width of the connections in the analysis is proportional to the number of recorded events, providing a representation of the most frequently used shipping routes (Fig. 2b).

The assortativity coefficient for both ecoregions and ports within Chile, as well as between ecoregions and ports in Chile and internationally, showed negative values, suggesting a tendency for nodes to connect with other nodes of different types (Table S5). Cramér’s V index indicated a strong association between ecoregions and ports (> 0.7, Table S5). Complementing these structural patterns, high similarity values were observed between the network and the environmental variability. For the ecoregions of Chile, a PSI of 0.842 was observed, while between the ecoregions of Chile and international, the PSI indicated a value of 0.772.

### Comparison between national and international ports

**Fig. 3:**
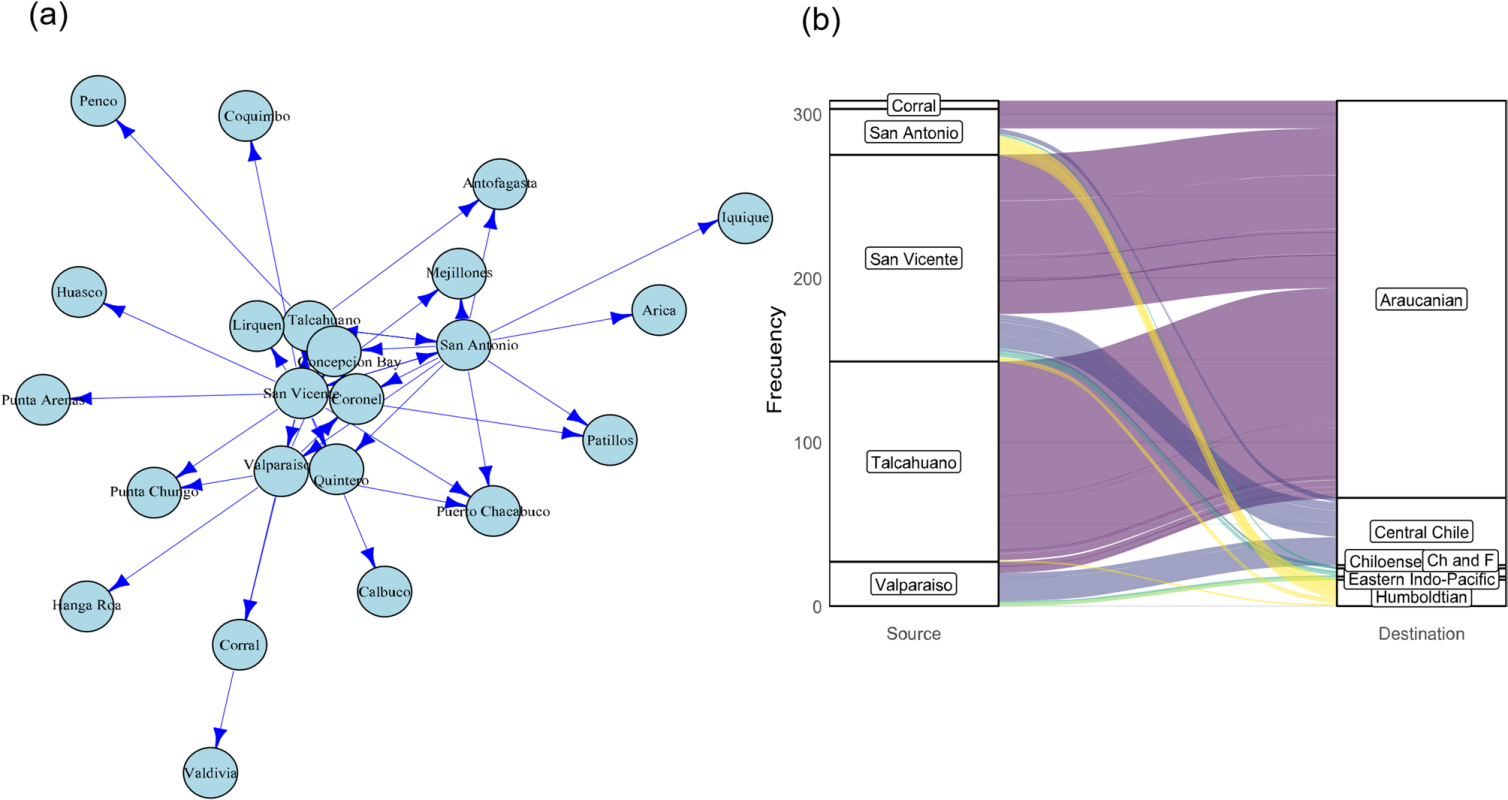
(**a**) Chilean Port Network. The size and density of the edges between the nodes represent the strength of connectedness. (**b**) Alluvial diagram relating the ports of origin and destination within Chile. The connection width is proportional to the number of events. Figures done using R (https://www.r-project.org).

The analysis of the national port network revealed that the ports of San Antonio, San Vicente, and Concepción Bay are the primary connection nodes within Chile, concentrating the highest vessel traffic and acting as strategic redistribution points for maritime traffic (Fig S1). These ports form a highly connected triangle, positioning them as key corridors for the exchange of NNS between the Central Chile, Araucanian, and Humboldtian ecoregions. Centrality indices confirmed their significance within the national network, recording the highest values for degree, closeness, and strength (Table S6). In contrast, secondary ports such as Corral, Mejillones, and Iquique showed lower levels of connectivity, indicating their limited role within the national port network. These patterns are graphically represented in the national port network (Fig. 3a) and the alluvial plot illustrating the connections among national ports (Fig. 3b).

**Fig. 4:**
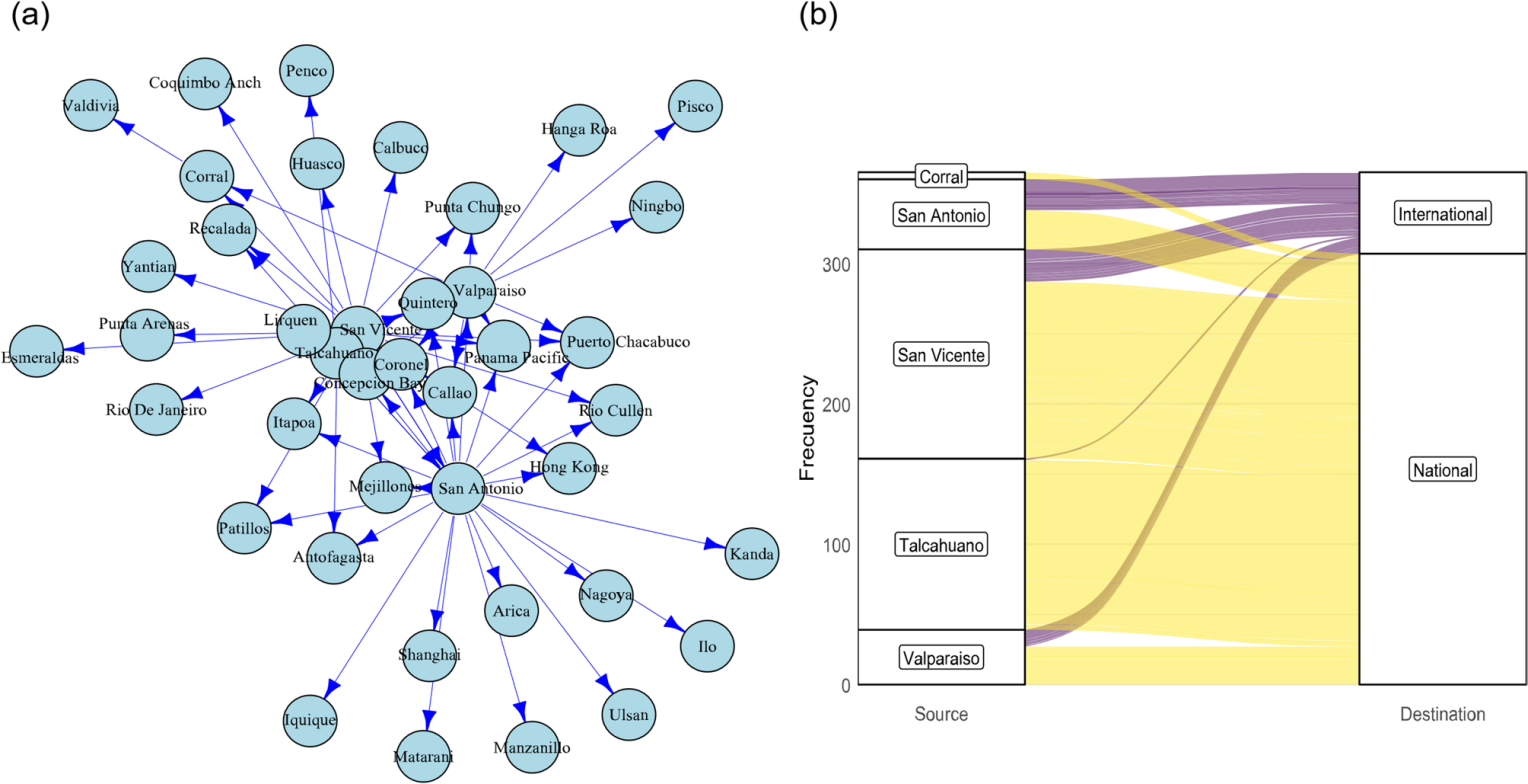
(**a**) Network of vessels between national and international ports. (**b**) Alluvial diagram relating the ports of origin (Chile) and destination outside Chile. The connection width is proportional to the number of events. Figures done using R (https://www.r-project.org).

At the international level, the ports of San Antonio and San Vicente once again stood out as the main connection nodes, establishing frequent routes to international hubs such as Manzanillo (Mexico), Panama, Shanghai, and Hong Kong. These ports not only maintain importance locally but also excel in metrics such as degree and strength within the international network (Table S7). Secondary ports, such as Corral and Mejillones play a limited role, primarily facilitating connections with destinations in the South Pacific, including Callao (Peru) and Esmeraldas (Ecuador). The international port connection network, stressing the relative importance of each node within the global network (Fig. 4a). Additionally, the alluvial plot revealed that the Corral, San Antonio and Valparaiso ports exhibit a higher flow towards international ports, while San Vicente and Talcahuano showed a greater flow towards national ports (Fig. 4b).

## Discussion

Our findings are consistent with patterns described by network-based studies in other regions of the world. At the global scale, ports with higher centrality, combined with similar temperature and salinity conditions, have been shown to act as key hubs for species transport across the maritime network^29^. Along the Australian coast, temporal models showed that ballast water discharges between environmentally similar ports increase the risk of propagule pressure and the establishment of NNS^30^. Together, this evidence supports that the combination of high centrality and environmental similarity, the same pattern detected among Chilean ecoregions, consistently increases the likelihood of NNS spread.

A particularly relevant result is the high environmental similarity between ports in the Biobío region and many other national and international ports. This similarity suggests that local conditions resemble those of source regions, increasing the likelihood that transported species will survive and establish. Previous studies have documented the presence of multiple established NNS in San Vicente port^21,22^. The Procrustes similarity index (PSI) yielded high values for both domestic (PSI = 0.842) and international connections (PSI = 0.772), indicating that environmental similarity may facilitate the establishment of NNS, particularly in highly connected areas^27^. This finding highlights the need to monitor the most connected nodes and their associated routes, demonstrating that combining network analysis with environmental similarity metrics is an innovative approach already validated in other marine regions^30^.

At the ecoregional scale, Central Chile and the Araucanian region stand out for their high centrality and connectivity, positioning them as key zones within the national network. Both handle intense maritime traffic and share strong environmental similarity, which may facilitate the spread of NNS. In contrast, the Humboldtian and Chiloense ecoregions exhibit lower connectivity, although they remain at risk due to their links with more interconnected regions. Internationally, San Antonio and San Vicente emerge as strategic hubs, maintaining frequent routes to global centers such as Panama and Shanghai. The observed network structure suggests that certain ports defined by their size, geographic position, and number of arrivals act as entry and redistribution points for biological vectors such as ballast water and biofouling.

An illustrative case is the ascidian *Asterocarpa humilis*, first detected in the sea chests of a tanker operating along the Antofagasta–Talcahuano route. The species is now established in mussel farming facilities approximately 2,000 km farther south^16^. Along this corridor, the model indicates high environmental similarity (Euclidean distance ≈ 5.4; Table S2), confirming that conditions at both ends are comparable. These metrics corroborate field observations and demonstrate the model’s ability to flag high-risk connections driven by port connectivity and ecological similarity.

Our findings also highlight the importance of intermediate-strength nodes: while major international hubs receive the highest number of arrivals, medium-sized ports can act as stepping stones when favorable environmental conditions coincide with frequent coastal traffic. Therefore, identifying potential entry points for NNS via vectors such as ballast water and biofouling requires the simultaneous consideration of both global and domestic fleets. However, it is important to note that this approach is limited by the lack of detailed trafficflow data for each port, which could improve the model’s accuracy. To increase the reliability of future analyses, further efforts should be dedicated to acquiring comprehensive traffic datasets.

This work highlighted the value of integrating network analysis with in-situ biological monitoring and port-environment assessments to prevent NNS introductions in Chile. Pinpointing the central nodes of San Antonio, San Vicente, and Concepción Bay provides a solid basis on which to prioritize management and enforcement at strategic locations. Collaboration among governmental agencies and non-governmental organizations is essential for implementing effective ballast-water and biofouling management systems, thereby enabling evidence-based oversight. Such alliances would bolster enforcement capacity and position Chile as a regional leader in marine conservation, aligning its policies with International Maritime Organization standards. In conclusion, network analysis is a powerful tool for preventing biological invasions: it identifies high-risk routes and key nodes, supports targeted inspections and surveillance, and informs regulatory updates on ballast-water management and hull cleaning. Because the approach is readily transferable, it offers a versatile framework for protecting marine biodiversity and mitigating ecological and economic impacts worldwide. Based on our findings, we recommend: (i) Strengthen the inspection and monitoring of compliance with regulations related to ballast water management and hull biofouling, with a focus on high-connectivity ports. (ii) Implement analyses that incorporate multidimensional variables, such as the network analysis used in this study, to enable such prioritization. (iii) Incorporate complementary regulations that ensure not only international biosecurity but also biosecurity between ecoregions within countries, especially in areas of high biodiversity and conservation value.

## Methods

**Fig. 5:**
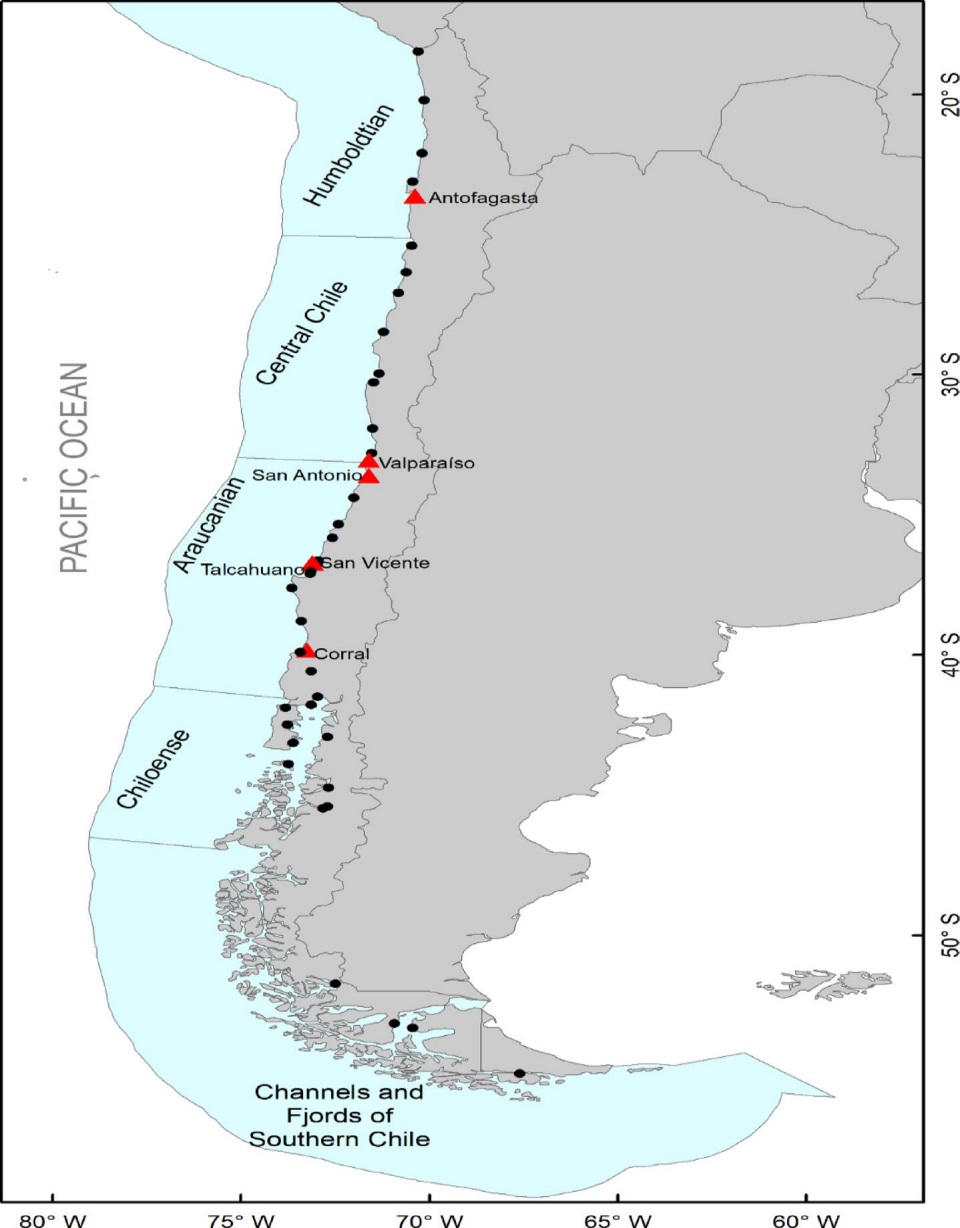
Map representing the ports and ecoregions used in this study. The black dots indicate ports present in Chile. Red triangles indicate ports studied, classified within the marine ecoregions (light blue): Humboldtian, Central Chile, Araucanian, Chiloense, and Channels and Fjords of Southern Chile. This figure was generated using ArcGIS 10.4.1. The map projection is WGS84 (www.esri.com/arcgis).

Maritime traffic data corresponding to the year 2023 were sourced from the MarineTraffic platform (https://www.marinetraffic.com). Six commercial ports in Chile were considered: Antofagasta, Corral, San Antonio, San Vicente, Concepción Bay (Fig. 5). Also, the port of origin and the type of vessels (e.g., general cargo, tankers, passenger, and fishing vessels) arriving at the six ports were recorded (See Fig. S1). These six ports were selected because of constraints in the availability of comprehensive and reliable data for additional ports; they are also critical nodes in both national and international maritime networks, whereas the remaining ports handle mainly domestic traffic and were therefore excluded. Network analyses were conducted using the ports of origin and destination together with the marine ecoregions in which each port is located, following Spalding et al.^31^. Edge weights were calculated to represent the frequency of connections between origin and destination ports, thereby constructing a weighted network in which each edge reflects the connection strength between nodes.

To assess the connectivity between ports and ecoregions of origin and destination, network analyses were used to calculate centrality indices: degree, closeness, betweenness, and strength^32,33^. Centrality indices quantify the importance of a node and how it influences the potential dispersion of organisms^34^. The degree indicates the number of direct connections it has within a network, determining the likelihood of a node being invaded or spreading the invader^34^. Closeness quantifies a node’s relationship with all other nodes, differing from the previous metrics by emphasizing the shortest average distance from a node to all others. Nodes with a shorter total distance to other nodes are more important for spreading NNS as they can connect with other nodes in fewer steps^34^. Betweenness indicates the importance of a node in the average path between node pairs, where nodes with higher betweenness can spread NNS faster and with higher probability. Finally, node strength reveals the magnitude of a node’s connection with others and is based on the strength of all connections of a specific node relative to others^32^.

We also calculated the degree of connection between nodes using the assortativity degree. Assortativity degree quantifies whether nodes with a high degree (i.e., high connectivity) connect with other highly connected nodes, ranging from − 1 to + 1. A positive assortativity degree indicates that well connected nodes tend to connect with other well-connected nodes, while negative values suggest that well-connected nodes tend to connect with poorly connected ones, providing information on network robustness^34^. Centrality indices and assortativity degree were calculated using the ‘igraph’ package^35^. Additionally, to evaluate the association between ports and/or ecoregions, we calculated the Cramer’s V index^36^. The index, ranging from 0 (no association) to 1 (perfect association), is derived from a corrected Chi-squared statistic and was calculated using the ‘oii’ package^37^.

To assess the relationship between network and environmental variability present in the ecoregions, we applied a Procrustes Similarity Index (PSI). From the networks we obtained the Euclidean distance matrix, and the environmental similarity matrix was calculated from the variables net primary productivity (NPP), pH, sea surface salinity (SSS) and sea surface temperature (SST) present in the ecoregions. Environmental data was obtained from Bio-Oracle 2.2^38^. The PSI takes a value between 0 and 1. Values close to 1 indicate that the two matrices are highly similar, whereas values close to 0 indicate that they are different. The calculations were performed with the ’MatrixCorrelation’ package^39^.

Finally, to assess the frequency of events from origin to destination ports, we evaluated this trend by ecoregion and port type using alluvial diagrams. Alluvial diagrams group observations of the same category and visualize them as flows through the considered set of characteristics^40^. These analyses were performed with the ‘ggalluvial’ package^41^. All analyses were performed in R 4.4.2^42^.

## Supplementary Information

Below is the link to the electronic supplementary material.

Supplementary Material 1

## Data Availability

The datasets generated and analysed during the current study are available in the Zenodo repository, 10.5281/zenodo.14779673.
