# Supplementary material for: Network-based risk assessment of ship-mediated dispersal of non-native species across Chilean and international ports
Source: Sci Rep. 2025 Aug 20;15:30482. doi: 10.1038/s41598-025-15482-y (PMC12365233; doi:10.1038/s41598-025-15482-y)
Supplement: Supplementary file 1 — Supplementary Material 1 [file 41598_2025_15482_MOESM1_ESM.docx]

**Supplementary Table**

Table S1. Centrality indices for vessel flow within Chilean marine ecoregions. Indices are shown as standardized z-scores

| **Ecoregion** | **Degree** | **Closeness** | **Betweenness** | **Strength** |
| --- | --- | --- | --- | --- |
| Araucanian | 4 | 0.014 | 1 | 51 |
| Central Chile | 4 | 0.030 | 1 | 34 |
| Chiloense | 2 | - | 0 | 5 |
| Eastern Indo-Pacific | 1 | - | 0 | 2 |
| Humboldtian | 1 | - | 0 | 16 |

Table S2. Pairwise Euclidean distances between ecoregions; lower distances denote higher environmental similarity.

|  | Araucanian | Central Chile | Humboldtian | Chiloense | Easter Island |
| --- | --- | --- | --- | --- | --- |
| Araucanian | 0 |  |  |  |  |
| Central Chile | 2.182 | 0 |  |  |  |
| Humboldtian | 5.444 | 3.267 | 0 |  |  |
| Chiloense | 2.589 | 4.769 | 8.033 | 0 |  |
| Easter Island | 8.578 | 6.397 | 3.149 | 11.164 | 0 |

Table S3. Centrality indices for vessel flows between Chilean marine ecoregions and international ecoregions. Indices are shown as standardized z-scores.

| **Ecoregion** | **Degree** | **Closeness** | **Betweenness** | **Strength** |
| --- | --- | --- | --- | --- |
| Amazonia | 1 | - | 0 | 2 |
| Araucanian | 16 | 0.008 | 9 | 99 |
| Central Chile | 8 | 0.009 | 1 | 46 |
| Central Kuroshio Current | 1 | - | 0 | 2 |
| Central Peru | 2 | - | 0 | 23 |
| Chiloense | 2 | - | 0 | 5 |
| East China Sea | 2 | - | 0 | 4 |
| Eastern Indo-Pacific | 1 | - | 0 | 2 |
| Humboldtian | 2 | - | 0 | 19 |
| Mexican Tropical Pacific | 1 | - | 0 | 1 |
| Panama Bight | 2 | - | 0 | 7 |
| Papua | 1 | - | 0 | 1 |
| Patagonian Shelf | 1 | - | 0 | 2 |
| Rio de la Plata | 1 | - | 0 | 2 |
| Sea of Japan | 1 | - | 0 | 1 |
| Southeastern Brazil | 1 | - | 0 | 6 |
| Southern China | 1 | - | 0 | 6 |

Table S4. Pairwise Euclidean distances between Chilean and international ecoregions based on environmental variables. Lower distances denote higher environmental similarity.

|  | Araucanian | Central Chile | Humboldtian | Central Peru | Southern China | Southeastern Brazil | Papua | Mexican Tropical Pacific | Central Kuroshio Current | Panama Bight | Chiloense | Patagonian Shelf | East China Sea | Sea of Japan/East Sea | Rio de la Plata | Amazonia | Easter Island |
| --- | --- | --- | --- | --- | --- | --- | --- | --- | --- | --- | --- | --- | --- | --- | --- | --- | --- |
| Araucanian | 0 |  |  |  |  |  |  |  |  |  |  |  |  |  |  |  |  |
| Central Chile | 2.182 | 0 |  |  |  |  |  |  |  |  |  |  |  |  |  |  |  |
| Humboldtian | 5.444 | 3.267 | 0 |  |  |  |  |  |  |  |  |  |  |  |  |  |  |
| Central Peru | 7.024 | 5 | 1.609 | 0 |  |  |  |  |  |  |  |  |  |  |  |  |  |
| Southern China | 11.470 | 9 | 6 | 4.950 | 0 |  |  |  |  |  |  |  |  |  |  |  |  |
| Southeastern Brazil | 9.869 | 8 | 4 | 3 | 4.122 | 0 |  |  |  |  |  |  |  |  |  |  |  |
| Papua | 15.307 | 13 | 10 | 8 | 4 | 6.3 | 0 |  |  |  |  |  |  |  |  |  |  |
| Mexican Tropical Pacific | 13.928 | 12 | 9 | 7 | 3 | 5 | 1.409 | 0 |  |  |  |  |  |  |  |  |  |
| Central Kuroshio Current | 8.828 | 7 | 4 | 2 | 3 | 2 | 6 | 5.141 | 0 |  |  |  |  |  |  |  |  |
| Panama Bight | 12.959 | 11 | 8 | 7 | 2 | 5 | 3 | 2 | 4.569 | 0 |  |  |  |  |  |  |  |
| Chiloense | 2.589 | 5 | 8 | 10 | 14 | 12 | 18 | 16 | 11 | 15.426 | 0 |  |  |  |  |  |  |
| Patagonian Shelf | 5.413 | 8 | 11 | 12 | 17 | 15 | 21 | 19 | 14 | 18 | 2.972 | 0 |  |  |  |  |  |
| East China Sea | 7.920 | 6 | 4 | 3 | 4 | 5 | 8 | 6 | 2 | 5 | 10 | 13.297 | 0 |  |  |  |  |
| Sea of Japan/East Sea | 1.143 | 3 | 6 | 8 | 12 | 11 | 16 | 15 | 10 | 14 | 2 | 5 | 8.650 | 0 |  |  |  |
| Rio de la Plata | 22.583 | 23 | 24 | 24 | 23 | 26 | 25 | 25 | 23 | 23 | 23 | 24 | 21 | 21.970 | 0 |  |  |
| Amazonia | 13.995 | 12 | 9 | 7 | 3 | 6 | 2 | 1 | 5 | 1 | 16 | 19 | 6 | 15 | 23.452 | 0 |  |
| Easter Island | 8.5786 | 6 | 3 | 2 | 4 | 1 | 7 | 6 | 2 | 6 | 11 | 14 | 4 | 10 | 25 | 6.609 | 0 |

Table S5. Network analysis results for each ecoregion and port type.

|  | Assortativity degree | Cramer 's V |
| --- | --- | --- |
| *Ecoregion* | | |
| National | -0.471 | 0.950 |
| National+International | -0.356 | 0.700 |
| *Port* | | |
| National | -0.09 | 0.735 |
| National+International | -0.131 | 0.752 |

Table S6. Centrality indices for vessel flows between Chilean ports. The indices are shown as standardized z-scores.

| **Port** | **Degree** | **Closeness** | **Betweenness** | **Strength** |
| --- | --- | --- | --- | --- |
| Antofagasta | 2 | - | 0 | 3 |
| Arica | 1 | - | 0 | 3 |
| Calbuco | 1 | - | 0 | 2 |
| Concepción Bay | 4 | - | 0 | 119 |
| Coquimbo | 1 | - | 0 | 1 |
| Coronel | 4 | - | 0 | 43 |
| Corral | 3 | 0.2 | 4 | 8 |
| Hanga roa | 1 | - | 0 | 2 |
| Huasco | 1 | - | 0 | 2 |
| Iquique | 1 | - | 0 | 1 |
| Lirquén | 2 | - | 0 | 43 |
| Mejillones | 2 | - | 0 | 7 |
| Patillos | 2 | - | 0 | 2 |
| Penco | 1 | - | 0 | 1 |
| Puerto Chacabuco | 3 | - | 0 | 3 |
| Punta Arenas | 1 | - | 0 | 2 |
| Punta Chungo | 2 | - | 0 | 5 |
| Quintero | 3 | - | 0 | 23 |
| San Antonio | 14 | 0.014 | 28 | 30 |
| San Vicente | 18 | 0.014 | 16 | 131 |
| Talcahuano | 9 | 0.012 | 4 | 143 |
| Valdivia | 1 | - | 0 | 5 |
| Valparaíso | 9 | 0.030 | 8 | 37 |

Table S7. Centrality indices for vessel flows between Chilean port and international port. Indices are shown as standardized z-scores.

| **Port** | **Degree** | **Closeness** | **Betweenness** | **Strength** |
| --- | --- | --- | --- | --- |
| Antofagasta | 2 | - | 0 | 3 |
| Arica | 1 | - | 0 | 3 |
| Calbuco | 1 | - | 0 | 2 |
| Callao | 3 | - | 0 | 23 |
| Concepción Bay | 4 | - | 0 | 119 |
| Coquimbo | 1 | - | 0 | 1 |
| Coronel | 4 | - | 0 | 43 |
| Corral | 3 | 0.2 | 4 | 8 |
| Esmeraldas | 1 | - | 0 | 1 |
| Hanga Roa | 1 | - | 0 | 2 |
| Hong Kong | 2 | - | 0 | 2 |
| Huasco | 1 | - | 0 | 2 |
| Ilo | 1 | - | 0 | 1 |
| Iquique | 1 | - | 0 | 1 |
| Itapoa | 2 | - | 0 | 6 |
| Kanda | 1 | - | 0 | 1 |
| Lirquén | 2 | - | 0 | 43 |
| Manzanillo | 1 | - | 0 | 1 |
| Matarani | 1 | - | 0 | 1 |
| Mejillones | 2 | - | 0 | 7 |
| Nagoya | 1 | - | 0 | 2 |
| Ningbo | 1 | - | 0 | 2 |
| Panama Pacific | 3 | - | 0 | 6 |
| Patillos | 2 | - | 0 | 2 |
| Penco | 1 | - | 0 | 1 |
| Pisco | 1 | - | 0 | 1 |
| Puerto Chacabuco | 3 | - | 0 | 3 |
| Punta Arenas | 1 | - | 0 | 2 |
| Punta Chungo | 2 | - | 0 | 5 |
| Quintero | 3 | - | 0 | 23 |
| Recalada | 2 | - | 0 | 2 |
| Rio Cullen | 2 | - | 0 | 2 |
| Rio de Janeiro | 1 | - | 0 | 2 |
| San Antonio | 26 | 0.009 | 56 | 52 |
| San Vicente | 27 | 0.009 | 24.5 | 154 |
| Shanghai | 1 | - | 0 | 2 |
| Talcahuano | 10 | 0.007 | 4.5 | 144 |
| Ulsan | 1 | - | 0 | 1 |
| Valdivia | 1 | - | 0 | 5 |
| Valparaíso | 13 | 0.022 | 14 | 49 |
| Yantian | 1 | - | 0 | 2 |

Figure S1. Heatmap of the number of vessels categorized by type and port.
